# Competing Conversations: An Examination of Competition as Intrateam Interactions

**DOI:** 10.3389/fpsyg.2019.00970

**Published:** 2019-05-03

**Authors:** Elsheba K. Abraham, Maureen E. McCusker, Roseanne J. Foti

**Affiliations:** ^1^Virginia Polytechnic Institute and State University, Blacksburg, VA, United States; ^2^Consortium Research Fellows Program, Alexandria, VA, United States

**Keywords:** constructive competition, organizational discourse analysis, social interdependence, team satisfaction, team performance, task conflict

## Abstract

Intrateam competition is an inherently social and interactional process, yet it is not often studied as such. Research on competition is mostly limited to studying it as an individual state and assumes that the resulting team outcomes are equivalent across different competition types. Often overlooked in competition research are the means through which competition can lead to constructive outcomes for the team. Constructive competition occurs when the primary motivation is not to win at the expense of others, but rather to make social comparisons and gain knowledge of relative competence. This study furthers insight into constructive competition by studying its interpersonal characteristics as it develops within a team, and its impact on task conflict, perceived performance, and team satisfaction. The conversations of 24 student project teams were recorded over 4 weeks and analyzed, operationalizing competition as an attempt to exert control and influence on the team. Each individual then provided sociometric ratings of perceived performance of each team member, and rated the level of task conflict and satisfaction of the team. The effects of competition on perceived performance and team satisfaction, both directly and indirectly through task conflict, were examined. Findings demonstrated a negative direct effect of competition on the range of perceived performance ratings, and a positive indirect effect of competition on team satisfaction as mediated through task conflict. The study broadens understanding on the construct of competition and underscores the positive implications competition can bring to the teams.

## Introduction

“*Great things in business are never done by one person; they’re done by a team of people.”*

– Steve Jobs

Regardless of the type of organization or the industry, a majority of companies today are converging toward team-based business models (McDowell et al., 2016). Teams enable organizations to diversify their range of expertise and skills, giving them the flexibility to adapt to the current market and maintain a competitive edge (Kozlowski and Bell, 2003). Prior teams researchers have found positive relationships between team effectiveness and organizational performance (e.g., Banker et al., 1996; Hamilton et al., 2003; Srivastava et al., 2006; Barrick et al., 2007). These findings align with what is intuitively known amongst employees; 91% of individuals across industries agree that teams are central to an organization’s success (Martin and Bal, 2015). Put simply, work teams are ubiquitous and are an essential part of our professional life. Thus, it is critical for organizations to have effective, efficient, and highly functioning teams.

Teams are characterized as a collection of two or more individuals who interact socially and perform interdependent tasks within an organizational context (Kozlowski and Bell, 2003). Team performance is not only a function of completing tasks assigned to the team, but also includes the results from team processes and individual effort (Katzenbach and Smith, 1993). Thus, how individuals interact with others can influence a team’s success (Marks et al., 2001). There is a level of social interdependence present within a team, as individuals share common goals and carry out actions that can influence the outcomes experienced by others (Stanne et al., 1999). Competition occurs if there is a negative relationship of goal attainment between two individuals – that is, if a goal sought by an individual clashes with goal achievement of someone else (Deutsch, 1949, 1962, 1969). Opposing goal structures induce individuals to act out in self-interest; it influences their interactions patterns and encourages actions that improve chances of obtaining personal goals at the expense of others, ultimately leading to competitive outcomes for the team (Johnson, 2003; Deutsch, 2011). This understanding between the conditions in a team and subsequent interactions that result in competition is the basic premise of the Social Interdependence Theory (Deutsch, 1949; Johnson, 2003).

As one of the major types of interdependencies observed in organizations (Tjosvold, 1986), competition has long been of focal interest for scholars studying team performance and states. Some of the earliest work can be traced back to the 1920s when Whittemore studied competition amongst workers in a printing task (Whittemore, 1924). Since then, research on how competition can influence the overall functioning of the team and subsequently team performance has expanded. The literature spans across domains from social and educational psychology (e.g., Julian and Perry, 1967; Johnson et al., 1979) to organizational conflict management (e.g., Alper et al., 2000; Lu et al., 2010) and sports psychology (e.g., Scanlan and Lewthwaite, 1984; Ntoumanis et al., 2007). Despite this, there is still some debate whether competition results in positive (Michaels, 1977; Young et al., 1993) or negative (Grossack, 1954; Kohn, 1992) outcomes. In fact, reviews of the research literature point to both (Miller and Hamblin, 1963; Johnson et al., 1981; Stanne et al., 1999).

We believe the lack of clarity of the team competition-outcome relationship stems from at least three issues, which we aim to address in this study. The first issue relates to how competition is operationalized. Different types of competition are often thought to be equivalent, which is problematic as the nature and outcomes from competition can vary according to competition type (Stanne et al., 1999). The second issue pertains to the measurement of competition as a construct. Deutsch (1949) highlights the dynamic, process-oriented nature of competition; it is a process of team interactions, influenced not only by the task but also by prior behaviors of team members that can impact future actions. However, competition is seldom studied as a process. Rather, it is most often measured through self-report, which provides only a single, often biased, snapshot of the competitive process. Third, there is a general disregard for the social context in which team interactional processes are embedded (Cronin et al., 2011). This is problematic, as competition is closely related to the interactions that occur from the social interdependencies present within a team (Deutsch, 1969).

Thus, the purpose of this study is to take a *process-oriented* approach to studying competition based on observable dyadic interactions within a team’s natural context. In doing so, we aim to obtain deeper insight into the specific nature of competition, how it influences outcomes such as perceived performance and team satisfaction, as well as the means through which these relationships emerge. This study contributes to the literature of intrateam competition, both methodologically and theoretically. Methodologically, we present a novel, process-oriented approach to capturing competition within a team, based in organizational-discourse analysis (Fairhurst and Uhl-Bien, 2012). Theoretically, we shed more clarity on the relationships between intrateam competition and team outcomes to better understand how and when competition is beneficial or detrimental for organizational units.

### Intrateam Competition

Competition can exist in many forms and levels – between units or collectives (interteam competition; Johnson and Johnson, 1991), between individuals (interpersonal competition; Deutsch, 1949), and between individuals in a collective (intrateam competition; Julian et al., 1966). Intrateam competition is understood as the encouragement of inter-individual competition and comparison between individuals in a unit (Ntoumanis et al., 2007), and it is commonly discussed in relation to goals (Deutsch, 1949). That is, interaction patterns between individuals vary as a function of perceived goal interdependence. Cooperative intrateam interactions occur when individual team members each possess interrelated goals and rely on each other to work toward shared goals; on the other hand, competitive intrateam interactions occur when individual goals are mutually exclusive and efforts are focused on increasing chances of attaining a personal goal (Deutsch, 1969, 2011).

Goals are fundamental regulators of human action (Locke et al., 1981) and thus are constant driving forces of behavior. In the context of teams, individuals learn to collaborate with one another to progress toward an overarching goal, such as successfully completing a team project. However, while team members work toward completing a team-level goal, they may also have personal goals they try to achieve concurrently (Brouwer, 2016). Thus, intrateam competition occurs when an individual in a unit perceives a negative outcome interdependence; that is, the progress toward one goal results in movement away from someone else’s goal (Deutsch, 2011).

#### Intrateam Competition as a Process

Researchers often study competition as an independent, stable construct divorced from the reality in which it is couched (Sommer, 1995). However, this deviates from how competition was originally conceptualized as a construct. In his seminal work on the topic, Deutsch (1949) identified the fundamental connection between competition and interpersonal relations, highlighting the importance of studying “the interactions between individuals, (and) the group process that emerges as a consequence of a cooperative or competitive social situation” (Deutsch, 1949, p. 1). Not only is competition a dynamic construct, but it also occurs within a team that is a dynamic entity in it of itself, where tasks, goals, and often members change frequently (Cronin et al., 2011).

To study competition accurately, researchers must align its operationalization with its conceptualization as a dynamic process (Kozlowski and Klein, 2000). While the sophistication of methodology and analytic techniques has vastly improved, utilization of these dynamic methods of capturing behavior is still lacking within the competition literature. The use of self-report to assess competition dominates, and most studies measure competition as a global construct and not within the context of the specific team (Gelfand et al., 2012). This is problematic since competition is dependent on various contextual factors that can vary across teams. Furthermore, self-report measures fail to capture the nature of interactions between individuals in an organization as they unfold over time (Knapp et al., 1988).

As interpersonal communication is at the core of small-group processes (Bonito and Sanders, 2011), measuring verbal interactions is one method of getting closer to understanding processes as they unfold in teams (Fairhurst and Uhl-Bien, 2012; Lehmann-Willenbrock and Allen, 2018; McCusker et al., 2018). This process-oriented measurement approach captures the social and interactional process mechanisms of competition. In the past decade, there has been a growing emphasis on studying interactions *in situ* to deepen our understanding on a variety of organizational constructs such as leadership (Wodak et al., 2011), employee attitudes (Meinecke et al., 2017), team coordination (Kauffeld and Lehmann-Willenbrock, 2012), and team diversity (Harvey, 2013). When intrateam competition occurs, interactions vary often as individuals try to balance maximizing individual performance relative to the group while ensuring the level of team performance (and thereby individual performance) is not negatively impacted (Miller and Hamblin, 1963). Hence, measuring competition as a process through interpersonal interactions enables proper emphasis on the social dynamics of intrateam competition. In this regard, one particularly valuable research strategy that could be applied is organizational discourse analysis (ODA; Putnam and Fairhurst, 2001; Grant et al., 2004). In ODA, verbal interactions between individuals are recorded or observed, and then coded and analyzed to uncover patterns of communication that reflect individual, dyadic and team phenomena. This process-oriented approach provides a means for better understanding of the full development of particular constructs instead of relying on snapshots based on an individual’s perception of what happened.

To the authors’ knowledge, there have been no other empirical studies within the organizational sciences that have studied intrateam competition through the lens of ODA, and more specifically, interpersonal interactions. Interpersonal dynamics carry a big impact on team outcomes, especially when participation from team members is necessary for a task (Tjosvold, 1986); hence, we seek to understand the team outcome implications for competition in the context of our study by observing intrateam interactions. The present study measures competition through *in situ* communicative interactions between team members as they worked together to accomplish a team task over time. As the aim of the study was to better clarify the impact of competition on team effectiveness, we focused on two of its most prevalent indicators in team literature: performance and team satisfaction (e.g., Gladstein, 1984; Costa, 2003).

### Team Outcomes of Competition

The research literature has found mixed results regarding the outcomes of competition on teams. Some research has found that competition has a positive impact. It encourages individuals to be more engaged with a task (Goldman et al., 1977) and to outperform their peers (Julian and Perry, 1967; Scott and Cherrington, 1974). However, the advantages associated with competition are typically more pronounced when the task can be completed independently and is routine (Deutsch, 1949; Erev et al., 1993), as competition enhances speed (Sommer, 1995; Beersma et al., 2003). Despite some of these positive findings regarding competition, the consensus is that aside from the specific conditions of a task that requires little collaboration between individuals, intrateam competition does not lead to advantageous team outcomes (Johnson et al., 1981; Stanne et al., 1999). Competition has been found to result in poorer performance for complex, and highly interdependent tasks in which two or more people are required to complete a task (Miller and Hamblin, 1963; Tjosvold, 1986; Stanne et al., 1999). Research also shows competition taints communication when individuals collaborate on a task (Deutsch, 1969, 2011), and increases hostile behavior and suspicion as individuals seek to preserve their personal goals (Deutsch, 1949).

Thus, intrateam competition has been found to result in both positive and negative outcomes for teams. While one explanation for this discrepancy may be the type of task, as discussed above, we believe an alternative explanation is the over-simplistic conceptualization of the construct of competition. There remains a fair amount of ambiguity regarding the conceptualization and operationalization of competition in the literature (Stanne et al., 1999). Nonetheless, there is now a growing body of research that investigates the different dimensions and forms of competition (Johnson and Johnson, 1974, 1991; Tjosvold et al., 2003, 2006). Competition can be classified into two types: *zero-sum competition*, where the winner-takes-all, and *constructive competition* (also known as appropriate competition), where winning is not given much importance (Stanne et al., 1999). Research on zero-sum competition dominates the field (Stanne et al., 1999; Tjosvold et al., 2003), yet both types of competition carry very different implications for the team. The lack of empirical work on constructive competition fails to reflect how commonly it occurs, from classrooms (Johnson and Johnson, 1974) to organizations (Tjosvold et al., 2003) as well to what it means for individuals and collectives. To fill this gap in the literature and heed calls to further develop theory on competition (Stanne et al., 1999), we focus this study on constructive competition and its impact on team processes and states.

#### Constructive Competition

A distinct characteristic of constructive competition distinguishing it from zero-sum competition is the relative weakness of negative outcome interdependence experienced (Stanne et al., 1999). In other words, although there is still an opposing goal structure between individuals, it is not the primary motivation to engage in competition. This phenomenon occurs when winning is not the top priority for individuals within the unit, the rules and process for winning is fair and specific, and everyone has a reasonable chance of achieving a specified goal (Johnson and Johnson, 1991; Stanne et al., 1999; Johnson, 2003; Tjosvold et al., 2003). Additionally, competition is constructive when individuals can monitor their progress by making comparisons of their performance relative to others. Social competition motivates individuals to expend greater effort and perform better than others in the team (Nicholls, 1984; Sommer, 1995); thus, these comparisons of competence, instead of winning, become the impetus for competing with others (Johnson and Johnson, 1974; Stanne et al., 1999).

Empirical research in this domain demonstrates that constructive competition leads to positive outcomes, even in situations or types of tasks where competition may not seem particularly advantageous. For example, in a study of Chinese managers and subordinates working in business organizations, Tjosvold et al. (2003) found that intellectually stimulating tasks were related to increased learning and self-efficacy when constructive competition was present. Additionally, perceiving an intrinsic motivation in the competitive rival who engages in competition due to enjoyment of the process itself can positively influence subsequent interactions. As such, there is an increase in constructiveness of competition experienced, as measured by task-related and affective-related benefits (Chang and Chen, 2012).

#### Competition and Perceived Performance

Project teams often must complete tasks that are highly interdependent, requiring constant communication and coordination to refine ideas and efforts (Mathieu et al., 2014). Intrateam competition may manifest as individuals attempt to gain status by dominating actions and influencing team decisions (Zhao, 2015). However, if the desire to achieve overall team success is prioritized, it can lead to effective collaboration with others in the team - building off of ideas and engaging in productive conversations where relevant information is shared with all (Deutsch, 1990). When there is a basis of cooperation that underlies interactions within the team, constructive competition is likely to occur and can motivate individuals to display the skills and knowledge needed to progress team effort and increase effectiveness in completing a task (Johnson and Johnson, 1991; Tjosvold et al., 2003). The increase in effort and effectiveness may spur individuals to engage further in social comparisons to assess their competence. Thus, team members would be more perceptive to the performance of their peers. Accordingly, we predict:

Hypothesis 1: Intrateam competition will have a negative direct effect on team members’ perceptions of each other’s performance.

#### Competition and Team Satisfaction

Team satisfaction is closely linked to intrateam processes. It is influenced not only by the nature of the task, but also from the interactions between team members (Van Der Vegt et al., 2001). When project teams work together on a task for an extended period of time, individuals have to account for not only the short-term advantages for engaging in competition but its implication in the long run. Long-term goals, such as establishing a rapport with team members through multiple project meetings, can increase the strength of an overall cooperative goal and subsequently encourage constructive competition (Tjosvold et al., 2003; Sheridan and Williams, 2011). Thus, competitive interactions in this context can lead to constructive outcomes to the team such as more positive relationships and desire to participate in the team (Tjosvold et al., 2003). There is still incentive to invest efforts in a task, but without the negative strain that is associated with competing solely to win (Wittchen et al., 2011). Additionally, when there is an unambiguous competitive climate, individuals can clearly assess their progress relative to others while knowing the nature of competition and what is needed to achieve their goal. Individuals are less likely to feel anxious or unhappy about team processes that occur (Johnson and Johnson, 1991). Hence, a sense of contentment with the team likely emerges.

Hypothesis 2: Team competition will have a positive direct effect on team satisfaction.

### The Role of Task Conflict

Though distinct concepts, intrateam competition and conflict are often confounded (Deutsch, 1969; Tjosvold, 1998). While intrateam competition can be conceptualized colloquially as a rivalry of goals, intrateam conflict can be understood as a clash in activities (Deutsch, 1969). The literature distinguishes between three types of team conflict: relationship, process, and task conflict (Jehn and Mannix, 2001). First, relationship conflict refers to discord and animosity between individuals within a team and is often associated with negative affect toward team members. Second, process conflict refers to disagreements about divvying up responsibilities and work among team members so that the task gets accomplished (Jehn, 1997). Third, task conflict refers to disagreements about opinions and ideas related to the task (Amason and Sapienza, 1997). Relationship and process conflict have consistently been found to be detrimental for both teams and individuals (Baron, 1991; Pelled, 1996; Jehn et al., 1999). However, task conflict has been found to be positively associated with a variety of team outcomes, including team satisfaction and performance (Hoffman and Maier, 1961; Korsgaard et al., 1995; Amason, 1996). Some researchers suggest the positive effect of task conflict on performance occurs because task conflict affords team members the opportunity to voice their opinions and discuss issues related to the task (e.g., Amason, 1996; Simons and Peterson, 2000; Wellman, 2013). Related are findings that teams are generally better able to manage task conflict, over relationship and process conflict. For example, in a study of 65 autonomous, newly formed teams, Behfar et al. (2008) found that the clear majority (71%) of teams that experienced consistent (and even increasing) levels of team performance and satisfaction over time effectively managed and resolved task conflict. In comparison, only one and 33% of the teams were able to address relationship and process conflict, respectively.

Despite the evidence that task conflict positively impacts team performance and satisfaction, little is known about the role of team competition in this relationship. Early research on teams and collectives suggest competition between groups of people drives interteam conflict, as proposed by Marx’s (1847) Conflict Theory and Realistic Conflict Theory (Sherif, 1958). At a more micro level, past research has found that competition is positively related to different forms of conflict (Brouwer, 2016). Competitive processes often induce behaviors that further perpetuate competitive interactions and facilitate conditions under which conflict emerges (Deutsch, 1994). As intrateam constructive competition is oriented toward addressing competing goals in order to push the task forward, it likely results in conflicting opinions about the task itself, or task conflict. Thus, if task conflict is positively associated with performance and satisfaction, and intrateam competition is positively associated with task conflict, it is likely that intrateam competition indirectly impacts team performance and satisfaction through task conflict.

Hypothesis 3a: Intrateam competition will have a negative indirect effect on team members’ perceptions of each other’s performance through task conflict.

Hypothesis 3b: Intrateam competition will have a positive indirect effect on team satisfaction through task conflict.

## Materials and Methods

### Sample and Procedure

Participants were 119 students (68% female) from three semesters of the same psychology course at a large, southeastern university. Participants were recruited from the classroom and received course credit for their participation in the study, and all students from all three semesters consented to participate. The course required a team project component, so all participants were randomly assigned to teams of four to six (*n* = 24 teams), stratified based on gender and academic major to ensure heterogeneity in teams. No participant reported being friends or acquaintances with any teammate prior to the first day of class. Teams were assigned a total of three different team projects, which they were to complete over the 12 weeks in the semester (4 weeks per project). The current study focused on the first project only, which entailed conducting an analysis of one team member’s previously held job. Teams worked together in class for 50-min once per week to complete the project. To minimize the amount of time participants spent working on the project outside of the classroom, participants were urged to complete as much of the project as possible in class.

To capture competitive interactions unfolding within teams, each team was recorded using audio recorders placed on each team’s table throughout the weekly class time devoted to working on projects. For coding purposes, prior to beginning project discussions, each team member identified his/herself verbally so that voices could be matched with names during the coding (discussed below). At end of the semester, all participants were sent a link to a questionnaire including relevant study variables and completed sociometric ratings of all other team members’ contributions to the projects.

This study was carried out in accordance with the recommendations of the Virginia Tech Institutional Review Board Policies and Procedures, following the ethical principles described in The Belmont Report and in applicable federal regulations. The protocol was approved by the Virginia Tech Institutional Review Board. All subjects gave written informed consent in accordance with the Declaration of Helsinki.

### Measures

#### Competition

Intrateam competition was operationalized as a pattern of communication between dyads in teams. Drawing from communication literature and ODA (e.g., Courtright et al., 1989) focusing on interpersonal interaction (interaction process analysis; Bales and Strodtbeck, 1951), the operational unit of analysis was a dyadic “interact” (Weick, 1979), or a reciprocal verbal communication from one person back to another (i.e., utterance from A to B and from B back to A).

#### Coding Interacts

All task-relevant verbal utterances representing a complete thought – or an attempt at a complete thought - were coded using an amended version of Fairhurst’s Relational Control Coding Scheme (1989). Non-task relevant communication was not coded. Each utterance included a code for the speaker, the recipient^1^ and type of utterance. Utterances could take on three different types: An assertion of control toward the recipient, or a *one-up* move (↑), an acquiescence of control toward the recipient, or a *one-down* move (↓), or a neutralization of control toward the recipient, or a *one-across* move (→). Assertion of control, or *one-up* moves, were the only types of interest for this study. As defined in previous relational control research (Fairhurst et al., 1995; de La Peña et al., 2012), an interact is a sequential pair of moves between dyad members. Thus, in this study, a competitive interact was defined as a sequential pair of *one-up* moves. The amount of intrateam competition was calculated as the percentage of competitive interacts occurring within the team over the course of the 4 weeks (i.e. number of competitive interacts/total interacts).

All verbal interactions were coded directly from the audio recordings, as opposed to transcriptions of the interactions, a process which has been found to more accurately capture interpersonal processes than coding based on transcriptions of data alone (Nicolai et al., 2010). All coding was conducted using INTERACT software (Mangold, 2010), a coding and statistical analysis program that provides a platform to develop codebooks, code behavioral data, and analyze interaction based data. Prior to coding, coders engaged in extensive training, practice and feedback, codebook refinement and tests of agreement. Formal training included the following: approximately 15 h of education on relational control coding and research (e.g., Courtright et al., 1989) and definitions of the codes included in the codebook; and approximately 25 h of coding practice and feedback, both independently together. Throughout this time, inclusion criteria for what to code, definitions of codes and how to code each utterance were refined.

Upon completion of training, pairwise agreement was calculated among all coders. As recommended for sequential behavioral observation research for this type of data (Bakeman et al., 2009; Lehmann-Willenbrock and Allen, 2018), agreement was calculated in multiple steps. The first step consisted of testing agreement on the unitization of utterances, or whether an utterance was “code-able.” The second step consisted of testing agreement on the content of relational control. Agreement was calculated using Light’s (1971) Kappa, which is an algebraic mean of all pairwise kappa values. Results showed adequate agreement across all three coding categories (*k* = 0.83 for unitization; *k* = 0.72 for relational control). To maintain levels of agreement, coders met bi-weekly throughout the coding process (∼20 weeks) to collectively discuss questions, concerns or idiosyncrasies. Following recommendations by Omerod and Ball (2017) to check for coding “drift” in agreement after substantial periods of time, the previous procedure was replicated upon completion of 2/3 of the audio recordings. Results of this coding drift study revealed higher Light’s Kappa values for all three coding categories (*k* = 0.77, -0.84).

#### Task Conflict

Task conflict was measured using Behfar et al.’s (2011) Task Conflict Scale. The 3-item scale, which measures an “awareness of differences in viewpoints and opinions about the team’s task” (Behfar et al., 2011, p. 128) has previously been found to be reliable (alpha = 0.84; Behfar et al., 2011). Responses are made on a five-point scale (*never* to *all of the time*) and include questions such as, “To what extent does your team argue the pros and cons of different options?”

#### Team Satisfaction

Team satisfaction was measured using Hackman’s (1988) Team Satisfaction Scale. The 3-item scale has previously been found to be reliable (alpha = 0.95; Cameron and Allen, 2014). Responses are made on a five-point scale (*strongly disagree* to *strongly agree*) and include questions such as, “Generally speaking, I’m very satisfied with the team.”

#### Perceived Performance

To measure team perceived performance, team members were asked to rate the degree to which each of their peers contributed to completing the project. Participants were asked to assign a rating from 70 to 115 to each team member, reflecting each team member’s effort in completing the project. Each participant was then assigned a score based on the average of the peers’ evaluations. To translate the individual level scores to the collective level, we calculated the range of team members’ scores. Thus, higher scores represented a larger discrepancy (range) in team members’ contributions, as rated by their team members, while lower team scores represented a lower discrepancy in this regard.

## Results

The means, standard deviations, reliability coefficients, and correlation coefficients of the variables of interest are reported in Table 1. All variables were aggregated and analyzed at the team level, as the primary purpose of this study is to examine how the interactions of dyads within a collective combine to impact the collective perceptions of team phenomena. This is also aligned with our theoretical hypotheses that focused on team outcomes. Due to the small sample size of the study, a more lenient criteria for significance is used, reporting all values that have a significance of *p* < 0.1 (Lavrakas, 2008). To facilitate the interpretation of our results, we also report the R^2^ effect sizes for the direct effects calculated from univariate regression analyses. According to Cohen et al. (2003), the effect size conventions for variance explained are 0.02, 0.13, and 0.26 for a small, medium, and large effect size, respectively. As for our indirect paths calculated from the mediation analyses, we report the standardized effect sizes for the indirect effects obtained from the STDYX standardization from Mplus. For single mediator-models, standardized effect sizes are generally unbiased and can be interpreted clearly (Miočević et al., 2018). However, due to the lack of clear guidelines on what constitutes a small, medium or large effect size of indirect effects (Miočević et al., 2018), we refrain from commenting on the size of the standardized indirect effects.

**Table 1:** Means, standard deviations, correlations, and reliability coefficients.

Variable	*M*	*SD*	1	2	3	4
(1) Competition	-1.31	0.36	–			
(2) Task conflict	8.88	1.06	0.53^∗∗^	(0.75)		
(3) Perceived performance	1.15	0.84	-0.39^∗^	-0.80	–	
(4) Satisfaction	12.8	1.32	0.20	0.48^∗^	-0.32	(0.72)

*N = 24. All variables are aggregated at team level. Numbers in the parentheses represent the Cronbach’s alpha values for reliability. ^∗^p < 0.1 and ^∗∗^p < 0.05.*

The bivariate relationships show that competition had a significant, negative relationship with perceived performance (*r* = 0.39 and *p* < 0.10). Given perceived performance was operationalized as the range of individuals’ peer rating scores, the negative relationship suggests that as competition increases within the team, peer performance ratings for each team member were more balanced. That is, more competition was associated with less discrepancy in the degree to which each person was perceived, on average, to contribute to the team. There was no significant correlation between competition and team satisfaction (*r* = 0.20, n.s.).

### Hypothesis Testing

**FIGURE 1:**
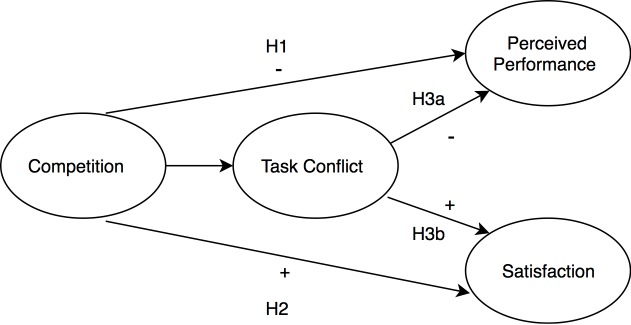
Hypothesized model of proposed direct and indirect effects of competition on perceived performance and team satisfaction.

**FIGURE 2:**
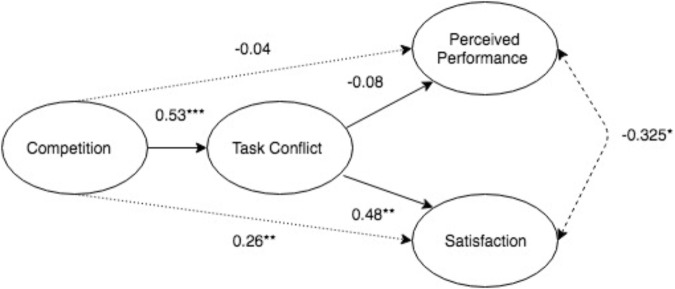
Results of hypothesized model of the direct and indirect effects of competition. Standardized path estimates are reported. The indirect effects are represented by the dotted arrows, while the covariance between outcome variables is represented by the dashed arrow. ^∗^*p* < 0.1, ^∗∗^*p* < 0.05, and ^∗∗∗^*p* < 0.001.

The hypothesized model, as pictured in Figure 1, was intended to be tested through a path model analysis conducted in Mplus (Version 8; Muthén and Muthén, 2017). As the hypothesized model was a just-identified or fully saturated model, model fit could not be assessed. Thus, the direct and indirect effects were tested separately to reduce the number of paths being estimated in the model and increase the degrees of freedom to examine model fit. Results for both models are depicted in Figure 2.

The direct effects of competition on each of the outcome variables were assessed through a simple linear regression. Results are reported in Table 2. Mirroring the correlations reported above, competition had a significant, negative effect on perceived performance (β = -0.39 and *p* < 0.10) and a medium effect size of *R*^2^ = 0.15. Thus, Hypothesis 1 was supported. However, competition had a no direct effect on team satisfaction (β = 0.20, n.s.) and a small effect size of *R*^2^ = 0.04, failing to support Hypothesis 2.

**Table 2:** Results of regression analysis testing direct effects of competition on outcome variables.

Variable	*B*	*SE*	β
**Competition**			
Perceived performance	-0.92^*^	0.46	-0.39
Team satisfaction	0.73	0.78	0.20

*N = 24. R^2^ for perceived performance = 0.15, R^2^ for team satisfaction = 0.04. ^∗^p < 0.10.*

Next, the model with the indirect effects was tested. Model fit was adequate, with χ^2^(2) = 6.08 (*p* = 0.05); SRMR = 0.095, CFI = 0.76. Significance of the indirect effects was evaluated using 95% confidence intervals from the bias-corrected estimates calculated from the 5000 bootstrapped samples drawn. Using bias-corrected bootstrapping estimates is recommended with small sample sizes, to obtain more accurate confidence intervals of the indirect effect (Efron and Tibshirani, 1994; Shrout and Bolger, 2002). Results showed that the indirect effect of competition on perceived performance was non-significant (-0.04; CI = -0.39 to 0.18; n.s.); hence, Hypothesis 3a was not supported. As for the indirect effect of competition on team satisfaction, competition had a positive, indirect effect on satisfaction through task conflict (0.26; CI = 0.06 to 0.48; *p* < 0.05). Thus, Hypothesis 3b was supported. Both the indirect and direct effects for the tested model are presented in Figure 2.

## Discussion

The vast majority of research on interpersonal competition focuses on its negative consequences for individuals and collectives (Tjosvold et al., 2003, 2006). This study, however, supports and contributes to a growing body of research finding that competition within teams can in fact have beneficial outcomes. Specifically, we found that the more that teams engaged in competitive interactions, the more equally they perceived their fellow team members’ performance. Furthermore, these findings showed that competition did not directly impact team satisfaction, but instead affected team satisfaction through task conflict. However, the indirect effect of competition was not significant for perceptions of performance.

### Contributions and Implications

We contribute to enriching both theoretical and empirical understanding of the constructive outcomes of competition and situate it within the broader literature of related constructs such as task conflict, performance, and satisfaction. Our findings highlight the positive effects of constructive competition. Teams benefit from competition when winning is not the main priority, competition is based upon clear and fair rules, each competitor has a comparable chance of winning, and there is opportunity to monitor performance relative to others. This study provides clearer insight into the impact of constructive competition within the natural constraints of a project team in a classroom.

The positive direct effect found between competition and perceptions of performance suggests that increased efforts to influence the team, such as with contributions of ideas or control over team-task processes, is advantageous for standardizing perceptions of performance across the team. This is an indication of cohesion within the team, as everyone is believed to have invested effort on the task; as a result, assessment of a team member’s performance is comparable with others within the team. Additionally, results demonstrate that competition and, in turn, task conflict are beneficial for teams, specifically in perceptions of satisfaction. This aligns our findings with past research that shows that effective task conflict management, characterized by open communication that is driven by facts and not emotions, leads to high team satisfaction (Behfar et al., 2008). Thus, when all individuals can contribute to the team and are engaged in constructive competition, an increase in intrateam interactions allows for a greater variety of viewpoints of the task to be discussed; this could ultimately lead to a better reflection of consensus with the team, and contribute to team satisfaction.

Unlike team satisfaction, we did not find an indirect effect of constructive competition on perceptions of performance through task conflict; however, results yielded a direct effect. While an indirect effect of competition on perceived performance was not found, relationships were in the hypothesized negative direction, suggesting the finding could, in part, be due to a low power to detect effects with the small sample of 24 teams. Alternatively, there could be other means through which competition impacts perceived performance. One plausible mechanism could be through achievement goals. Murayama and Elliot (2012) suggest an opposing processes model, in which they argue that the competition-performance relation is mediated by two different types of achievement goals: *performance-approach* goals where individuals are motivated to outperform their peers, and *performance-avoidance* goals where individuals try not to perform poorly relative to others. In the context of the present study, an increase in competition heightens awareness of others’ performance as individuals make social comparisons, which could potentially prompt individuals to adopt a particular type of achievement goal (Murayama and Elliot, 2012). Congruence between similar types of achievement goals within a team could potentially explain a convergence in perceptions in performance. Given the inherent relationship between individual goals and competition, goal congruence with regard to approach/avoidance goals presents a potential opportunity for fruitful research.

Additionally, our findings carry relevant implications beyond this study, as individuals may often find themselves in contexts where informal comparisons of performance are made instead of being in a winner-take-all situation. Our research coincides with other studies that have found the positive effects that constructive competition can have on teams. Constructive competition is generally found to increase performance across academic, workplace, and sports settings when competitors have a similar chance of succeeding (Worrell et al., 2016). For example, in a study examining intrateam competition amongst athletes, teams that had coaches who emphasized constructive competition during practices had better performance and greater enjoyment of athletes (Harenberg, 2014). In a completely different setting, Büki (2013) also demonstrated the positive effects of constructive competition in a group of Brazilian immigrants. Constructive competition facilitated integration between the immigrants and the host country, hence enabling the immigrant group to succeed in acculturation.

Moreover, this study contributes methodologically to the literature on constructive competition. First, most competition research either experimentally manipulates competition within teams or measures the construct using a self-report questionnaire, captured at some point after the fact. However, to measure competition as a process, and not a state (Deutsch, 1969), we drew from communication and discourse literature (Fairhurst and Uhl-Bien, 2012) to introduce a novel way to measure and thus operationalize constructive competition, which is better aligned with its conceptualization as a social process.

Second, collecting *in situ* data of newly formed teams working together in a naturalistic setting over time adds to the external validity of our findings. In modern-day organizations, competitive environments rarely resemble the exaggerated zero-sum competition often manipulated or assumed in the typical operationalization of competition. Given that organizations are gravitating toward more team-based, flat hierarchical structures, project-based teams are forced to work together to accomplish tasks. This environment certainly does not preclude intrateam competition from emerging, as humans’ drive for status, power and control of resources underlie modern day intrateam social competition (Hays and Bendersky, 2015). However, such teams inherently require some degree of coordination and cooperation in order to complete projects and tasks. This reflects constructive competition (Tjosvold et al., 2006) or “coopetition” (Brandenburger and Nalebuff, 1996), which is more representative of the informal competition present in today’s organizations. Our research design reflects this type of team setting and allows for measurement of a more realistic form of social competition; thus, provides a more direct application to organizations and practice.

Third, by operationally defining social competition as a dyadic verbal interaction using discourse, we treat competition not as a quality of a person or a situation, but rather as social processes manifested in the way a pair of individuals behave toward each other in context. We realize the limitations of aggregating dyadic level interactions to the team level (e.g., competition may have been primarily enacted by only one or two dyad members); however, with the exception of a few (e.g., Zhao, 2015), research does not tend to operationalize competition as the basic dyadic process that it is. We see this as an opportunity to provide novel insights into social competition, and by association, constructive competition at a relational level, as well as heed calls to advance dyadic level research in the organizational sciences (Krasikova and LeBreton, 2012).

### Limitations and Future Research

As with any research, this study is certainly not without its limitations. First, while data in this study were collected over the course of 4 weeks, team sample size prevented the ability to test longitudinal hypotheses. Thus, competition data were aggregated over the course of 4 weeks. Given the paucity of research on competition over time, research capturing the emergence process of competition within teams as it unfolds over time is a fruitful avenue for future research. Researchers have suggested that interactional process dynamics are often characterized by positive and negative spirals that can escalate or diminish particular dyadic phenomena (DeRue and Ashford, 2010) and often result in affective contagion (Kelly and Barsade, 2001). Thus, a better understanding of how teams vary on trajectories of competition can shed light as to how and why competition develops, changes, and is managed within different types of teams.

Second, due to how the data were coded, we were unable to clearly distinguish between specific types of social competition that transpired among team members. For example, Zhao (2015) distinguished among three types of interpersonal competition behaviors, each with different intentions: the intention to convey superiority over another with regard to competence, participation, and connection to others. While our codebook was intended to capture relational control as opposed to behavioral intent, developing codebooks that provide more a more fine-grained understanding of competitive interacts can provide a deeper understanding into interpersonal control manifests and unite disparate literature on the topics.

Another coding limitation was that all communication directed to the team in its entirety, as opposed to a specific individual, was excluded from analyses. This type of communication that is not directed to any particular individual in the team (e.g., talking to the room) has presented a challenge for small group researchers. While we recognize that control can be exercised toward all team members, the nature of an interact, as initially conceptualized by Weick (1979), is dyadic; thus we chose to eliminate all communications directed toward the team as a whole. We did, however, run the same analyses including team recipients and found similar results, so we feel justified in making this more conservative decision.

Despite these limitations, we believe our study lays a strong foundation for advancing the science of constructive competition. Given the relative novelty of the construct, there exist ample opportunities for advancing theoretical understanding. Particularly important, we believe, is research that focuses on additional cultural and contextual factors that might influence the relationships we uncovered in this and future research. As most of the research is conducted in laboratory/classroom settings and in North America (Tjosvold et al., 2003), identifying the contextual boundary conditions under which constructive competition unfolds is a critical condition for generalizability.

Aside from task conflict, another contextual factor that may mediate the effect of constructive competition on team performance is the degree to which a team is collectively or individualistically oriented. Due to the emphasis of constructive competition on fairness and equal chances of winning, the output of effort invested in a task should not be associated with a comparable amount of strain (Wittchen et al., 2011). However, individualistic teams that prioritize self-interest are likely driven by aversive competition (i.e., desire not to lose) as they anticipate the potential social consequences of losing; hence, they are more susceptible to strain when in competition (Wittchen et al., 2011). On the other hand, members in collectivistic teams who identify more strongly as a team than as separate individuals are likely to prioritize advancing the team. Hence, competition enhances task performance while any concerns about interpersonal issues or hostility that may arise from competitive processes are suppressed (Zhang et al., 2014). It is possible, then, that the saliency of an individual’s identity compared to the team’s identity influences how constructive competition is perceived. Those who adopt an individualistic mindset may emphasize winning more than those with a collectivistic perspective. It would be interesting to extend research by investigating the impact of different combinations of individualistic and collectivistic team members (e.g., homogenous collectivistic/individualistic; heterogenous), as one orientation could have more influence on team outcomes than the other.

In terms of potential moderators, one construct of interest is an organization’s level of distributive justice, which refers to the perceived fairness of the ratio of outcomes received to efforts invested (Folger, 1977). Individuals perceive distributive justice when rewards received are in proportion to the amount of work put in and that ratio is comparable to the outcome/effort ratio of others in the organization. Because past research has established that behaving fairly is positively associated with constructive competition (Tjosvold et al., 2003) and satisfaction (McFarlin and Sweeney, 1992), it may be that what makes competition constructive versus destructive is the degree to which it is perceived as fair. If the “rules” are clear and specific, and everyone has a comparable likelihood of winning (i.e., fair), then competition is likely to be constructive, leading to healthy task (not relationship) conflict and positive outcomes for the unit. Further research ought to investigate the role of perceived fairness in relationships between constructive competition and team outcomes. Another moderator to consider is perceived task complexity. Research shows that perceived task complexity increases intrateam performance when zero-sum competition is present; however, this relationship is limited to instances when the demands of the highly complex tasks do not exceed the ability of the individual to complete it (Brouwer, 2016). In the context of constructive competition, it is worth examining if task complexity would have a similar limiting effect on performance. Perhaps the opportunity to engage in comparisons of performance within the team could provide additional motivational resources to complete the task, thereby reducing the ceiling effect that task complexity could have on performance.

The area of constructive competition is rich with additional research questions, such as its temporal and social complexity. Previous research demonstrates that constructive competition encourages the desire to continue collaborating within the same team even after a task is completed (Tjosvold et al., 2003). Employing longitudinal research methods would allow us to examine how constructive competition and its impact on team outcomes changes over time. Additionally, although this study was purposefully conducted on project teams with no formal leadership, the presence of a formal hierarchy may impact the competition process. There is an extensive body of research supporting organizational conflict management, but the role of the leader in the conflict management and competition process is much in need of clarification (Behfar et al., 2011). Perhaps authentic leaders, who are highly self-aware and encourage positive self-development (Avolio and Gardner, 2005), can facilitate transparency within teams and thereby encourage the constructive effects of competition. Avenues for future research can include investigating the impact an individual’s authentic leadership style has on team outcomes of constructive competition. Finally, both relationship and process conflict are pertinent to social and task processes that happen in a team (Jehn and Mannix, 2001). As both types of conflict are negatively associated with performance and relationship conflict with satisfaction (Jehn, 1997), it would be interesting to investigate if that trend is maintained if constructive competition occurs in a team.

## Conclusion

The current study advances the study of competition and team outcomes in several ways. First, it is one of only a handful of studies to examine intrateam competition as an inherently social and interactional process. Second, it uses methodologies from interpersonal communication to measure team member’s verbal interactions. The benefit of studying actual verbal markers of a phenomenon such as competition between team members is that the obtained data are closer to the phenomena of interest, both conceptually and methodologically. Finally, our results highlighted the difference effects competition can have on team outcomes. In sum, this study shows that team competition is observable and impactful at the micro-level of team interaction processes. We hope that our findings will inspire future process research on competition and team dynamics.

## Ethics Statement

This study was carried out in accordance with the recommendations of the Virginia Tech Institutional Review Board Policies and Procedures, following the ethical principles described in The Belmont Report and in applicable federal regulations, with written informed consent from all subjects. All subjects gave written informed consent in accordance with the Declaration of Helsinki. The protocol was approved by the Virginia Tech Institutional Review Board.

## Author Contributions

All authors collaborated to review the literature, design the study, conceptualize the model, and edit the manuscript. EA led the efforts to write the manuscript and organize the team. MM led the data collection and analysis efforts. RF provided the extensive feedback and contributed to writing the introduction and discussion.

## Conflict of Interest Statement

The authors declare that the research was conducted in the absence of any commercial or financial relationships that could be construed as a potential conflict of interest.

## Footnotes

1 Utterances directed toward the team as a whole were removed from the analyses, as an interact requires a response from the recipient, and a team in its entirety cannot provide a response.
